# Identification of Host Cellular Protein Substrates of SARS-COV-2 Main Protease

**DOI:** 10.3390/ijms21249523

**Published:** 2020-12-15

**Authors:** Márió Miczi, Mária Golda, Balázs Kunkli, Tibor Nagy, József Tőzsér, János András Mótyán

**Affiliations:** 1 Department of Biochemistry and Molecular Biology, Faculty of Medicine, University of Debrecen, 4032 Debrecen, Hungary; miczimario@med.unideb.hu (M.M.); golda.maria@med.unideb.hu (M.G.); kunkli.balazs@med.unideb.hu (B.K.); tozser@med.unideb.hu (J.T.); 2 Doctoral School of Molecular Cell and Immune Biology, University of Debrecen, 4032 Debrecen, Hungary; 3 Department of Applied Chemistry, Faculty of Science and Technology, University of Debrecen, 4032 Debrecen, Hungary; nagy.tibor@science.unideb.hu

**Keywords:** COVID-19, coronavirus, SARS, SARS-CoV-2, main protease, 3CL protease, NetCorona, SSHHPS, cleavage site identification, cleavage site prediction, host protein cleavage

## Abstract

The novel severe acute respiratory syndrome coronavirus 2 (SARS-CoV-2) is the causative agent of coronavirus disease-19 (COVID-19) being associated with severe pneumonia. Like with other viruses, the interaction of SARS-CoV-2 with host cell proteins is necessary for successful replication, and cleavage of cellular targets by the viral protease also may contribute to the pathogenesis, but knowledge about the human proteins that are processed by the main protease (3CLpro) of SARS-CoV-2 is still limited. We tested the prediction potentials of two different in silico methods for the identification of SARS-CoV-2 3CLpro cleavage sites in human proteins. Short stretches of homologous host-pathogen protein sequences (SSHHPS) that are present in SARS-CoV-2 polyprotein and human proteins were identified using BLAST analysis, and the NetCorona 1.0 webserver was used to successfully predict cleavage sites, although this method was primarily developed for SARS-CoV. Human C-terminal-binding protein 1 (CTBP1) was found to be cleaved in vitro by SARS-CoV-2 3CLpro, the existence of the cleavage site was proved experimentally by using a His_6_-MBP-mEYFP recombinant substrate containing the predicted target sequence. Our results highlight both potentials and limitations of the tested algorithms. The identification of candidate host substrates of 3CLpro may help better develop an understanding of the molecular mechanisms behind the replication and pathogenesis of SARS-CoV-2.

## 1. Introduction

A novel severe acute respiratory syndrome coronavirus 2 (SARS-CoV-2) was identified in December 2019 as the causative agent of coronavirus disease-19 (COVID-19) that occurred first in Wuhan, Hubei province, China [1]. According to the data that were reported to the World Health Organization up to 10 December 2020, the global SARS-CoV-2 pandemic was associated with >68.16 million confirmed cases of infections and >1.55 million virus-related deaths worldwide (https://covid19.who.int). 

In general, viruses rely on the host machinery for the efficient infection and for the completion of the replication cycle, furthermore, changing expression profiles of host genes and interactions with the host proteins can also help the virus to evade the immune reaction after the infection, as it was observed in the case of SARS-CoV and SARS-CoV-2 infection, as well [2,3,4,5]. It is known that SARS coronavirus infection can influence multiple tissues or organs, including the respiratory system [6], coagulation system [7,8], gastrointestinal tract [9], or nervous system [10]. Numerous interacting partners of SARS-CoV-2 proteins have already been identified [3], but the detailed function and proteolytic targets of SARS-CoV-2 in the host cells are still understudied, however, various symptoms may be connected in part with the destruction of host proteins.

The genome of SARS-CoV-2 codes for multiple non-structural proteins (nsp) including two cysteine proteases, a papain-like protease (nsp3, PLpro), and a 3-chymotrypsin-like protease (nsp5, 3CLpro, or main protease), this last one is responsible for most of the processing of the viral polyprotein. Both SARS-CoV and SARS-CoV-2 3CL proteases consist of three domains. Domain I and II contain antiparallel β-barrels, while domain III has a helical arrangement. The active site comprises His41 and Cys145 catalytic residues [11,12,13]. 

**Figure 1:**
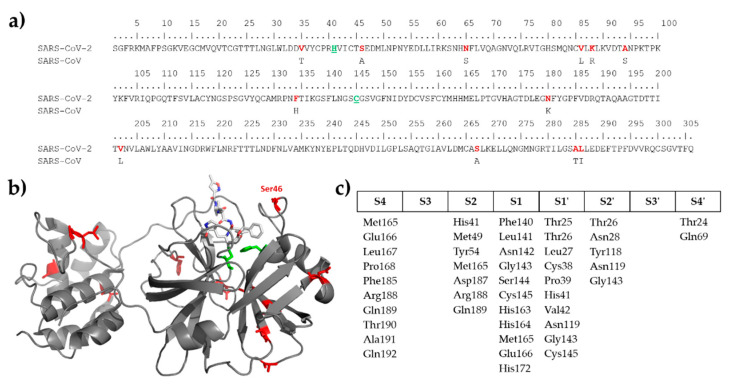
Comparison of SARS-CoV and SARS-CoV-2 3CL proteases. (**a**) Sequence alignment of the proteases is shown, only those residues are represented for SARS-CoV 3CLpro that are different as compared to SARS-CoV-2 3CLpro. Active site residues are green and underlined, residues that differ in the two proteases are red. (**b**) Schematic structure of SARS-CoV-2 3CLpro based on its X-ray crystal structure (6LU7.pdb). Catalytic residues (His41 and Cys145) are highlighted by green color, the residues that are different in SARS-CoV and SARS-CoV-2 3CL proteases are shown by red. Ser46 residue is also highlighted. Inhibitor bound to the active site is also shown by sticks. (**c**) Compositions of substrate binding subsites are shown based on literature data [11,12,13,16], subsite nomenclature is shown according to Schechter and Berger [15].

SARS-CoV and SARS-CoV-2 3CL proteases share high sequence identity (96%) [14] and differ only in few residues (Figure 1a), including the Ser46 (SARS-CoV-2 3CLpro numbering) which serine residue is located in the proximity of the active site of the enzyme (Figure 1b) but is not involved in the formation of any substrate binding subsite (Figure 1c). The substrate-binding cleft is located between domain I and II, and the substrate-binding subsites show high conservation [11,12,13]. Each amino acid side chain of the substrate (P4-P4’) which fit in a successive subsite of the enzyme (S4-S4’) is named according to the notation of Schecter and Berger [15]. The S4 site of the protease is a shallow hydrophobic site, while S3 enables binding of a wide range of residues, including hydrophobic (e.g., Val), polar (e.g., Thr), or basic (Arg, Lys) residues, because P3 residue is exposed to the solvent. S2 and S1 are deep sites, S2 shows a preference for hydrophobic P2 residues (Leu, Phe, Val) of autoproteolytic cleavage sites of the polyprotein, while S1 pocket specifically binds P1-Gln residue. The relatively shallow S1′ site mainly binds Ser or Ala residues, while the deep and hydrophilic S2′ site can accept a wide variety of the residues even a large Lys. Similar to P3, the P3′ residue is also exposed to the solvent, thus specific interactions are not formed with the protease at this site, and the shallow hydrophobic S4′ site also can bind various residues [11,12,13]. The high conservation of substrate binding subsites implies that efficient inhibitors may target a wide range of CoV 3CL proteases [13], and the specificity of SARS-CoV-2 3CLpro may be highly comparable with that of SARS-CoV.

The autoproteolytic cleavage site sequences of SARS-CoV and SARS-CoV-2 3CL polyproteins have already been described [17,18], but only a few cleavage sites were identified in host target proteins. It has been reported that SARS-CoV 3CLpro can cleave cellular V-ATPase G1 in vitro [19], and A549 human lung carcinoma cells overexpressing SARS-CoV 3CLpro showed down-regulated NF-κB production [20], the decreased NF-κB protein level may possibly be a consequence of the proteolytic processing of NF-κB by SARS-CoV 3CLpro. Based on the high-confidence interaction of SARS-CoV-2 3CLpro, histone deacetylase 2 (HDAC2) was also identified as a candidate target, and the catalytically inactive protease was found to interact with tRNA methyltransferase 1 (TRMT1), as well [3]. To date, only a single in vitro study has been reported in which an LC-MS based N-terminomics approach was applied to identify host targets of SARS-CoV and CoV-2 3CLpro by incubating the recombinantly expressed enzymes with cell lysates of lung and epithelial cells. Numerous host targets have been identified, the obtained cleavage site preferences which were derived from in vitro proteomic analyses revealed a high preference for P1-Gln, P2-Leu, and P1’-Gly/Ala/Ser residues [21].

The in silico methods are useful tools for the prediction of cleavage site sequences, such tools were designed for some viral proteases, e.g., for human immunodeficiency virus proteases (HIVcleave webserver) [22], picornaviral proteases (NetPicoRNA v. 1.0 webserver) [23], Group IV viral proteases [24,25], and an algorithm has also been developed for SARS-CoV 3CLpro (NetCorona 1.0 webserver) [26]. The identification of short stretches of homologous host-pathogen protein sequences (SSHHPS) was also used successfully to determine cleavage sites of Zika virus and Venezuelan equine encephalitis virus (VEEV) proteases in multiple human target proteins [24]. This method is based on the principle that host proteins may also contain such sequences that are identical with cleavage site sequences of viral polyproteins, therefore, may be potentially targeted by the viral protease. The NetCorona webserver was developed based on multiple cleavage site sequences of coronavirus polyproteins and is applicable for the prediction of potential cleavage sites of SARS-CoV 3CLpro, thus can be used for the identification of proteolytic targets and for inhibitor design, as well [26]. This algorithm was applied previously to predict cleavage sites in the nucleocapsid protein of porcine epidemic diarrhea virus (PEDV) 3CLpro [27], in the equine coronavirus polyprotein [28], or in human protein targets of SARS-CoV 3CLpro while developing the method [26]. In the case of SARS-CoV-2 3CLpro, glutathione peroxidase 1, selenoprotein F, and thioredoxin reductase 1 were proposed to be host substrates by using in silico algorithms [29], but the results were not validated in vitro. These proteins were not identified in the recently reported proteomic analysis as substrates of SARS-CoV-2 3CLpro [21], although, in vitro identification of host targets in additional cell types remain to be performed. Therefore, the application of in silico methods may aid the identification of proteolytic targets, and results of in silico analyses can be correlated with those of in vitro measurements to assess the reliability of predictions, which are widely used in the computational drug design. 

Accordingly, in this study, we aimed to apply SSHHPS analysis and the NetCorona 1.0 webserver to predict SARS-CoV-2 3CLpro cleavage sites. BLAST analysis was used to identify SSHHPS in human proteins, while NetCorona v. 1.0 webserver was applied for the prediction of cleavage probabilities. Structures of potential targets were also investigated to determine surface accessibilities of the predicted cleavage sites. Experimental approaches, including the design and use of His_6_-MBP-mEYFP recombinant protein substrates (MBP, maltose-binding protein; mEYFP, monomeric enhanced yellow fluorescent protein) were also applied to prove susceptibility for processing by SARS-CoV-2 3CLpro.

## 2. Results

### 2.1. Comparison of SARS-CoV and SARS-CoV-2 Protease Cleavage Sites

**Figure 2:**
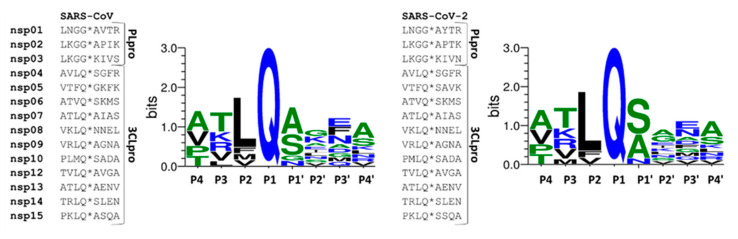
Comparison of SARS-CoV and SARS-CoV-2 cleavage site sequences. Sequences are shown for PLpro and 3CLpro based on literature data [15,16]. Cleavage positions are shown by asterisks, the sequences enclosed by the lines were used to prepare sequence logos. P4-P4’ substrate residues are numbered according to the nomenclature of Schechter and Berger [15].

First, we compared the autoproteolytic cleavage site sequences of SARS-CoV and SARS-CoV-2 3CLpro and found that the recognition sites closely resemble each other (Figure 2). Similar to SARS-CoV [21,30], SARS-CoV-2 3CLpro cleavage sites also contain a conserved Gln residue in the P1 position, and there are hydrophobic (Leu, Phe, or Val) and small aliphatic residues (mainly Ser or Ala) in P2 and P1’ positions, respectively. 

Both the identical binding site compositions (Figure 1) and high similarity of 3CLpro cleavage sites in the viral polyproteins (Figure 2) implied that SARS-CoV and SARS-CoV-2 3CL proteases share similar substrate profiles. Accordingly, the NetCorona v. 1.0 webserver which has been developed primarily for the prediction of SARS-CoV 3CLpro cleavage sites [26] was assumed to be potentially applicable to predict SARS-CoV-2 3CLpro cleavage sites, as well. 

### 2.2. Testing NetCorona 1.0 Webserver for Prediction of SARS-CoV-2 3CLpro Cleavage Sites

First, we tested whether NetCorona 1.0 algorithm is suitable for the identification of autoproteolytic cleavage sites within the SARS-CoV-2 polyprotein. As was expected, no NetCorona score was predicted for the cleavage sites of PLpro (nsp1, nsp2, and nsp3) because these sites are different from the consensus pattern of 3CLpro. Cleavage sites of 3CLpro were identified successfully by the webserver (Table 1), only the nsp5 site resulted in a score being slightly below the threshold, indicating that 87% sensitivity of the method [26] may be a limiting factor of prediction. The results implied that the NetCorona 1.0 webserver can be potentially applied to predict cleavage sites of SARS-CoV-2 3CLpro.

### 2.3. Identification of Host Targets by SSHHPS Analysis and NetCorona Prediction

The NetCorona algorithm was found to be an effective tool for the prediction of those cleavage sites within the full-length polyprotein sequences which show the consensus pattern (Table 1), no additional cleavage sites were identified in the polyprotein. 

We assumed that other methods that are based on the similarities of sequence motifs may also be applicable for cleavage site identification. Such a method is the SSHHPS analysis of which prediction potential has already been proved in the case of Group IV proteases [24,25]. We applied this method to find candidate targets of SARS-CoV-2 3CLpro, the SSHHPS were identified in human proteins by BLAST analysis using autoproteolytic cleavage site sequences of SARS-CoV-2 polyprotein as input (Table 1).

The results of SSHHPS analyses are shown in Table S1 for all cleavage sites of SARS-CoV-2 polyprotein. Numerous human proteins were found to contain such a site that is similar to the autoproteolytic cleavage sites of the polyprotein, highest similarities were obtained e.g., for C-terminal-binding protein 1 and 2 (CTBP1 and CTBP2), dihydropyrimidinase-related protein 2, protein tyrosine kinase 6 (PTK6), acetylcholinesterase (ACHE), protocadherin 19, JNK1/MAPK8-associated membrane protein, or obscurin proteins (Table S1). 

**Figure 3:**
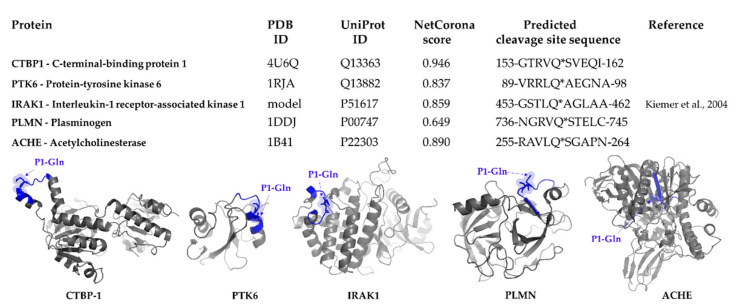
Putative SARS-CoV-2 3CLpro cleavage sites in some selected human target proteins. For the selected proteins, PDB and UniProt identifiers are shown, the scores and cleavage sites predicted by NetCorona 1.0 webserver are also indicated. Predicted score for IRAK1 was reported previously [26]. The structures of the proteins are also shown (grey), the predicted cleavage sites are highlighted (blue), arrows show the P1-Gln residues.

SSHHPS analysis showed a high similarity of a sequence motif of human PTK6 protein (^89^VRRLQ*AEGNA^98^) with that of the viral polyprotein (nsp9, TVRLQ*AGNAT). Accordingly, this site was identified by NetCorona 1.0 webserver with a relatively high probability, indicating that PTK6 contains a putative cleavage site of 3CLpro (Figure 3).

Human CTBP1 protein was also predicted to contain a sequence motif (^373^ELNGAAYRYP^382^) which is similar to the nsp1 site of SARS-CoV-2 polyprotein (ELNGG*AYTRY). The relatively high score obtained for this site by SSHHPS analysis indicated that CTBP1 may also be a proteolytic target. However, the identified cleavage site is likely to be a cleavage site of PLpro, the putative target sequence does not resemble the consensus pattern of 3CLpro cleavage sites and contains no glutamine in the P1 position. Despite this, the sequence of CTBP1 was analyzed by the NetCorona 1.0 webserver, as well (Figure S1). As it was expected, the single motif (^373^ELNGAAYRYP^382^) of CTBP1-identified by SSHHPS analysis-was not predicted as a putative cleavage site of 3CLpro, but interestingly the prediction revealed a putative 3CLpro cleavage site in CTBP1 (^153^GTRVQ*SVEQI^162^), which was not identified by SSHHPS analysis based on similarity with nsp4-15 cleavage sites. 

These results implied that the SSHHPS analysis may also be potentially applicable for the identification of the cleavage sites of PLpro, however, testing prediction potential in the case of PLpro was out of the scope of this study. NetCorona 1.0 webserver is applicable only for the prediction of 3CLpro sites.

### 2.4. Selection of Targets for In Vitro Investigation

Out of the possible targets identified by in silico sequence analyses, we selected CTBP1 and PTK6 for further investigation. These proteins were identified as candidate substrates of SARS-CoV-2 3CLpro using NetCorona 1.0 webserver, as well (Figure 3). Interleukin-1 receptor-associated kinase 1 (IRAK1) was predicted previously to be potentially cleaved by SARS-CoV 3CLpro [26], thus it was also selected for testing its proteolysis in vitro. We decided to include IRAK1 in this study in order to prove that potential targets of SARS-CoV 3CLpro may be accessible for cleavage by SARS-CoV-2 3CLpro, as well. Furthermore, to our best knowledge, cleavage of IRAK1 by SARS-CoV or SARS-CoV-2 3CLpro has not been proved experimentally to date. Interestingly, the cleavage site in IRAK1 was not identified by SSHHPS analysis, however, it was predicted with a high score by the NetCorona algorithm (Figure 3). This may highlight a limitation of SSHHPS analysis and implies that the number of potential targets may depend on the settings of the BLAST search.

It has already been described that COVID-19 caused by SARS-CoV-2 infection may lead to coagulation disorders and increased risk of venous thromboembolism [7]. Therefore, we investigated whether some human plasma proteins that may be susceptible to proteolysis by SARS-CoV-2 3CLpro. The sequences of these proteins were analyzed only by the NetCorona algorithm, SSHHPS were not identified by BLAST analysis in these proteins. Human plasminogen (PLMN) and plasminogen activator inhibitor 2 (PAI2) were identified as candidate substrates, while fibrinogen, plasminogen activators, and plasminogen activator inhibitor 1 (PAI1) were predicted to contain no putative 3CLpro cleavage site (Table S2). A higher score was obtained for PLMN as compared to PAI2, therefore, we selected PLMN for testing cleavage in vitro (Figure 3).

Identification of some plasma proteins (PLMN, PAI2) as potential targets by the NetCorona webserver implied that SSHHPS analysis alone may be not sensitive enough for the high throughput identification of protease substrates, however, these methods are based on different approaches for cleavage site identification, and the number of identified sites may depend on BLAST settings (e.g., length of query sequence). Additionally, we assumed that structural contexts of the putative cleavage sites need to be considered, therefore, accessibilities of target regions were also determined. A similar in silico approach has already been applied for the identification of potential cleavage sites in host selenoproteins and enzymes of glutathione synthesis [29], but neither proteomic [21] nor specifically targeted analyses proved cleavages of these targets in vitro to date. Interestingly, PAI2 was identified as a substrate of SARS-CoV and hCoV-NL63 3CLpro, as well, while cleavage of PLMN was not detected by a proteomic analysis [21].

To investigate whether the candidate substrates are sensitive towards proteolysis by SARS-CoV-2 3CLpro in vitro, we selected CTBP1, PTK6, IRAK1, and PLMN recombinant proteins because of the potential cleavage sites of these proteins were found to be exposed to the surface (Figure 3). The putative cleavage site in acetylcholinesterase (ACHE) was found to be buried in the structure, therefore, ACHE was excluded from further analysis. Example of ACHE proved the importance of structural analysis of candidate substrates: the possible target sequences may be inaccessible for proteolysis even the high probability of cleavage that was implied by sequence-based prediction (e.g., by NetCorona v. 1.0).

### 2.5. In Vitro Cleavage of Recombinant Proteins by SARS-CoV-2 3CLpro

**Figure 4:**
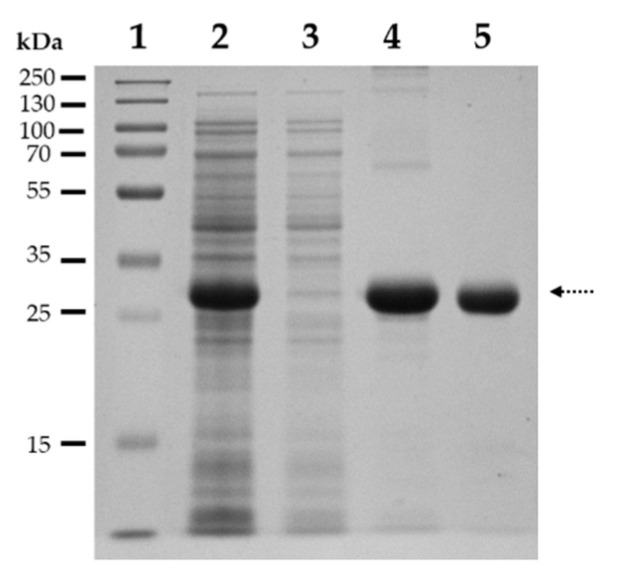
Purification of SARS-CoV-2 3CLpro. Figure shows a Coomassie-stained gel image, after SDS-PAGE analysis of samples: (1) Molecular weight standard; (2) Soluble lysate; (3) Ni-NTA affinity chromatography, flowthrough; (4) Ni-NTA affinity chromatography, eluate fraction of His_6_-SARS-CoV-2 3CLpro; (5) ion-exchange affinity chromatography, eluate of untagged SARS-CoV-2 3CLpro. Dotted arrow shows SARS-CoV-2 3CLpro, which has a slightly lower molecular weight than the His_6_-tagged enzyme.

For in vitro cleavage reactions we used untagged SARS-CoV-2 3CLpro. The protease was expressed in BL21(DE3) cells fused to an N-terminal His_6_-tag and then purified by Ni-NTA affinity chromatography. After the enzymatic removal of His_6_-tag using Factor Xa, the untagged enzyme was further purified by ion-exchange chromatography. The purity of the enzyme was assessed by SDS-PAGE (Figure 4).

**Figure 5:**
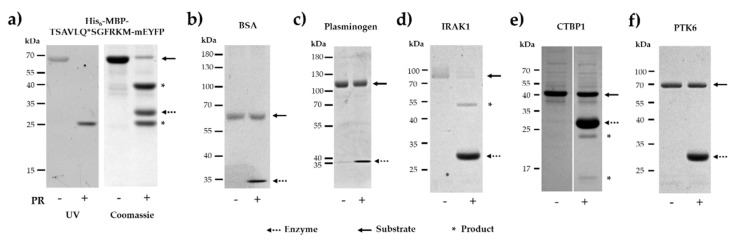
Cleavage of recombinant proteins by SARS-CoV-2 3CLpro. After cleavage reactions by SARS-CoV-2 PR, reaction mixtures were analyzed by SDS-PAGE. Uncleaved proteins and SARS-CoV-2 PR are shown by continuous and dashed lines, respectively, while asterisks indicate cleavage products. (**a**) Cleavage of His_6_-MBP-TSAVLQ*SGFRKM-mEYFP. After in-gel renaturation, the full-length substrate and SGFRKM-mEYFP cleavage product were visualized UV illumination, and the gel was stained with Coomassie dye, as well. (**b**) Cleavage reaction of recombinant BSA. (**c**) Cleavage reaction of recombinant plasminogen. (**d**) Cleavage of reaction recombinant IRAK1. (**e**) Cleavage reaction of recombinant PTK6. (**f**) Cleavage reaction of recombinant CTBP1. Gel images are representatives of ≥2 parallel experiments.

Cleavage reactions were performed by SARS-CoV-2 3CLpro to investigate the susceptibility of the selected human proteins for proteolysis (Figure 5). Additionally, a His_6_-MBP-mEYFP recombinant protein containing a natural cleavage site of SARS-CoV-2 3CLpro (nsp4, TSAVLQ*SGFRKM) was also designed and applied as a positive control substrate in the in vitro cleavage reactions. The NetCorona score obtained for the recombinant substrate was identical to the value calculated for the AVLQ*SGFR cleavage site of the polyprotein (Table 1). As was expected, the His_6_-MBP-TSAVLQ*SGFRKM-mEYFP fusion protein substrate was cleaved very efficiently by the protease. The substrate and cleavage products were separated by denaturing SDS-PAGE and then detected in the gel using UV transillumination, which indicated successful in-gel renaturation of mEYFP (Figure 5a).

For negative control, we applied bovine serum albumin (BSA) as a substrate of the untagged enzyme. Neither SSHHPS analysis nor NetCorona webserver predicted SARS-CoV-2 3CLpro cleavage sites in BSA (Table S2), in agreement with this we found that BSA was not processed by the protease (Figure 5b).

PLMN was also predicted to contain a putative cleavage site, but we observed no processing (Figure 5c). However, the NetCorona score obtained for the putative cleavage site was above the threshold (0.5) but was below the highest probability range (0.8–1.0) (Figure 3).

IRAK1 was identified previously as a candidate target of SARS-CoV 3CLpro [27], but its processing by a coronavirus 3CLpro has not been proved to date, therefore, IRAK1 was also subjected to proteolysis. We observed almost complete turnover of IRAK1 upon cleavage by SARS-CoV-2 3CLpro (Figure 5d), proving that IRAK1 is a proteolytic target of SARS-CoV-2 3CLpro. In this study, this protein was not newly identified as a candidate substrate, thus, was not further investigated in vitro.

CTBP1 protein was found to be processed but we observed lower turnover as compared to IRAK1 (Figure 5e). The appearance of cleavage products implied processing of CTBP1, we assumed that this cleavage occurs within the cleavage site identified by the NetCorona v. 1.0 webserver (^153^GTRVQ*SVEQI^162^). In order to prove the existence of cleavage between 157th and 158th residues, processing of CTBP1 was further investigated, as it is described later.

**Figure 6:**
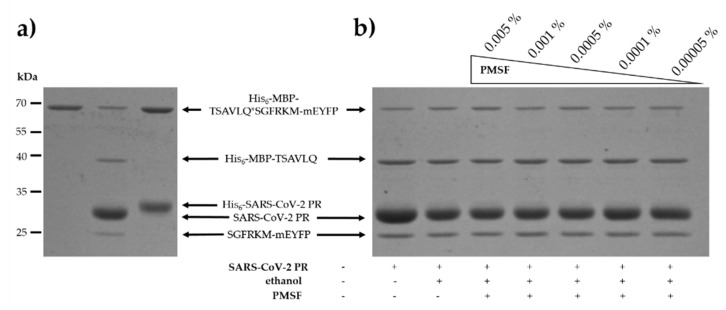
Effect of N-terminal His_6_ tag and PMSF on SARS-CoV-2 3CLpro activity. (**a**) In cleavage reactions we used His_6_-MBP-TSAVLQ*SGFRKM-mEYFP as a substrate of His_6_-tagged and untagged forms of SARS-CoV-2 3CLpro. (**b**) The effect of PMSF on the cleavage of His_6_-MBP-TSAVLQ*SGFRKM-mEYFP was studied, using untagged protease. PMSF was dissolved in ethanol and was applied in 0.005–0.00005% (*m/v*) concentration range. Uncleaved substrate is shown in figure part (**a**). Gel images are representatives of ≥2 parallel experiments.

PTK6 was identified as a potential target both by SSHHPS analysis and NetCorona prediction, but we did not detect its processing by SARS-CoV-2 3CLpro (Figure 5f). It is important to note that the recombinant PTK6 was supplied in a buffer containing phenylmethylsulfonyl fluoride (PMSF) which is known to be able to effectively inhibit numerous serine proteases (including chymotrypsin and trypsin). Therefore, to exclude the possibility that the processing of PTK6 was impaired by PMSF, we investigated its effect on SARS-CoV-2 3CLpro activity. We found that PSMF does not inhibit the processing of His_6_-MBP-TSAVLQ*SGFRKM-mEYFP recombinant substrate, even at 0.005% (*m/v*) final concentration (Figure 6b). This implied that PMSF present in the stock solution cannot prevent proteolysis and proved that PTK6 is not a proteolytic target of SARS-CoV-2 3CLpro.

The effect of the N-terminal His_6_-tag on the activity of 3CLpro was also tested, and the His_6_-tagged enzyme was unable for processing the recombinant substrate (Figure 6a). This in agreement with the findings of Grum-Tokars et al. who revealed a dramatic decrease of SARS-CoV 3CLpro activity upon addition of N- or C-terminal affinity tags [32].

**Figure 7:**
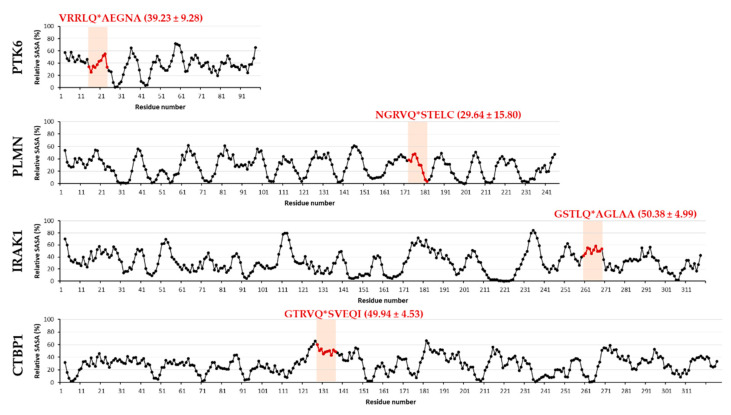
Analysis of surface-accessible surface areas. Relative surface-accessible surface area (SASA) values (%) determined for all atoms in PTK6, PLMN, IRAK1, and CTBP1, and were averaged for every position in a 5-residue window. Values for P5–P5’ residues are highlighted by red, the average-of-average and SD values calculated for P5–P5’ sites are shown in brackets. Residue numbering starts in each case from the first residue of the protein in the coordinate file.

In order to investigate the possible causes of why we observed no proteolysis in the case of some candidate targets, we further analyzed the structures and compared the accessibilities of the putative cleavage sites (Figure 7). The comparison of solvent-accessible surface areas of PTK6, PLMN, IRAK1, and CTBP1 structures showed that P5–P1 and P1’–P5’ residues may have relatively lower accessibility in PTK6 and PLMN, respectively. We assume that relatively lower solvent accessible surface areas (SASA) of these sites may prevent efficient binding and cleavage of the substrate. In contrast, the average values obtained for P5–P5’ residues are more comparable in CTBP1 and IRAK1 proteins and show relatively higher overall accessibility of the putative cleavage site, however, a threshold was not determined. We assumed that the relatively lower cleavage efficiency of CTBP1 may be caused in part by the accessibilities of P2’–P5’ residues of ^153^GTRVQ*SVEQI^162^ site which are located in an α-helix, while the entire target site of IRAK1 is located in a loop region. The relatively higher accessibilities of cleavage sites are in agreement with the susceptibilities of CTBP1 and IRAK1 for proteolysis in vitro (Figure 5) and indicate that probabilities of cleavage sites predicted by the NetCorona webserver need to be interpreted by considering surface accessibilities of putative sites, as well. Our result highlights that determination of apparent accessibilities of cleavage sites in the protein structures is not sufficient enough (Figure 2); in agreement with the results of Taylor and Radding [29], we also suggest the detailed determination of structural characteristics, especially the calculation of numerical SASA values (Figure 7) for more reliable cleavage site prediction.

### 2.6. Identification of Cleavage Position in CTBP1 and in His_6_-MBP-mEYFP Substrates

We designed and prepared His_6_-MBP-mEYFP recombinant fusion proteins which were used as substrates of SARS-CoV-2 3CLpro. A His_6_-MBP-TSAVLQ*SGFRKM-mEYFP substrate contained the natural nsp4 cleavage site of SARS-CoV-2 polyprotein, while a His_6_-MBP-REGTRVQ*SVEQIRE-mEYFP protein contained that cleavage site sequence which was identified CTBP1 by NetCorona algorithm (^153^GTRVQ*SVEQI^162^).

**Figure 8:**
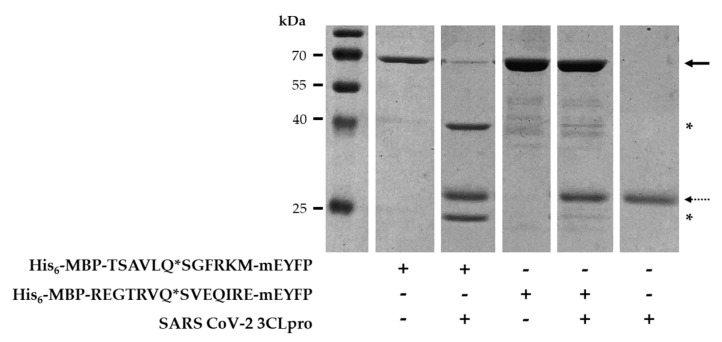
Cleavage of recombinant fusion protein substrates by SARS-CoV-2 3CLpro. His_6_-MBP-mEYFP substrates containing TSAVLQ*SGFRKM (SARS-CoV-2 nsp4) or REGTRVQ*SVEQIRE (CTBP1) cleavage site sequences were digested with SARS CoV-2 3CLpro. After SDS-PAGE, the gel was stained with Coomassie dye. Uncleaved proteins and SARS-CoV-2 PR are shown by continuous and dashed lines, respectively, while asterisks indicate cleavage products. Gel images are representatives of ≥ 2 parallel experiments.

As it was expected, His_6_-MBP-TSAVLQ*SGFRKM-mEYFP was processed by SARS-CoV-2 3CLpro (Figure 5a), and we observed proteolysis of His_6_-MBP-REGTRVQ*SVEQIRE-mEYFP protein as well. The substrate turnover was lower as compared to His_6_-MBP-TSAVLQ*SGFRKM-mEYFP which implies lower cleavage efficiency for the CTBP1 cleavage site (Figure 8). This is in contrast with the obtained cleavage probabilities, as a higher NetCorona score was obtained for the CTBP1 cleavage site (0.946) as compared to the nsp4 site (0.891). This observation implied that the cleavage probabilities predicted purely based on protein sequence may show no strong correlation with cleavage efficiencies, indicating that it is important to validate the results of in silico predictions in vitro.

**Figure 9:**
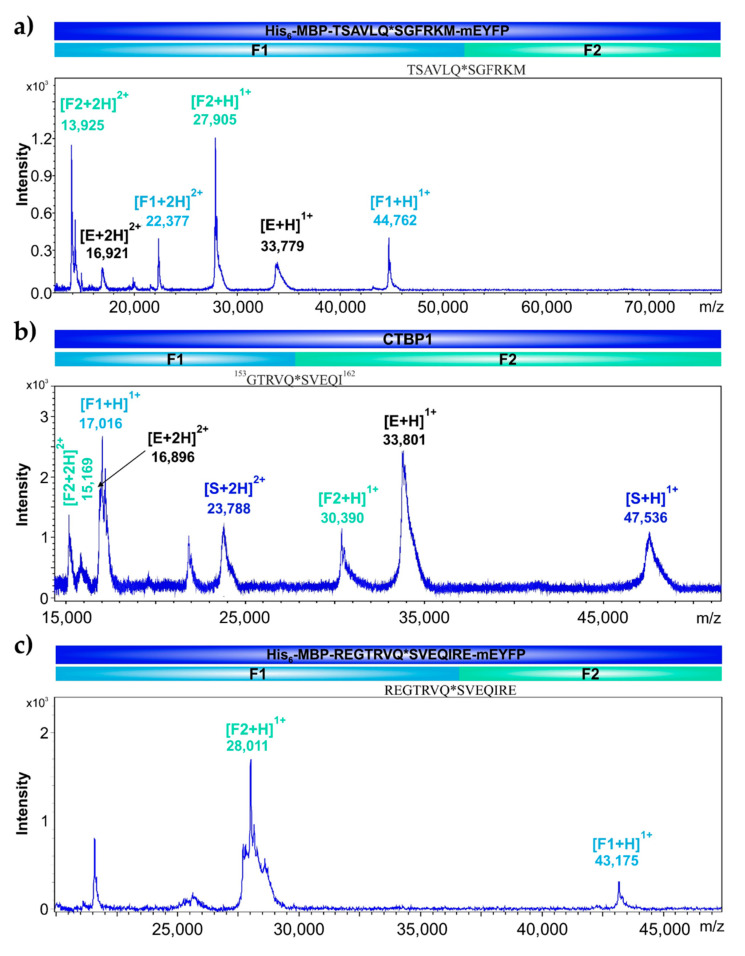
Determination of cleavage sites by MALDI-TOF MS. In cleavage reactions His_6_-MBP-TSAVLQ*SGFRKM-mEYFP (**a**), recombinant CTBP1 (**b**), and His_6_-MBP-REGTRVQ*SVEQIRE-mEYFP proteins (**c**) were used as substrates. Molecular weights (Da) of SARS-CoV-2 3CLpro are labeled by black, values for the full-length substrates are shown by dark blue color, while the proteolytic fragments are colored by light blue and green, respectively. Cleavage site sequences are also shown, asterisks indicate cleavage position.

The reaction mixtures were analyzed by matrix-assisted laser desorption/ionization time-of-flight mass spectrometry (MALDI-TOF MS) and the molecular weights of cleavage fragments were determined for the identification of cleavage positions. As was expected, the recombinant His_6_-MBP-mEYFP substrate representing the nsp4 cleavage site sequence of the polyprotein (TSAVLQ*SGFRKM) was cleaved within the incorporated sequence at the desired position (Figure 9a). After the cleavage of recombinant CTBP1 protein, the analysis of cleavage fragments implied that the full-length protein is cleaved at the predicted site (^153^GTRVQ*SVEQI^162^) (Figure 9b), and the recombinant His_6_-MBP-mEYFP substrate containing the same cleavage site was also cleaved at the same predicted position (Figure 9c). These results proved that the fluorescent substrates are suitable for fluorimetric assay.

### 2.7. Comparison of Cleavage Efficiencies of SARS-CoV-2 and CTBP Cleavage Sites

**Figure 10:**
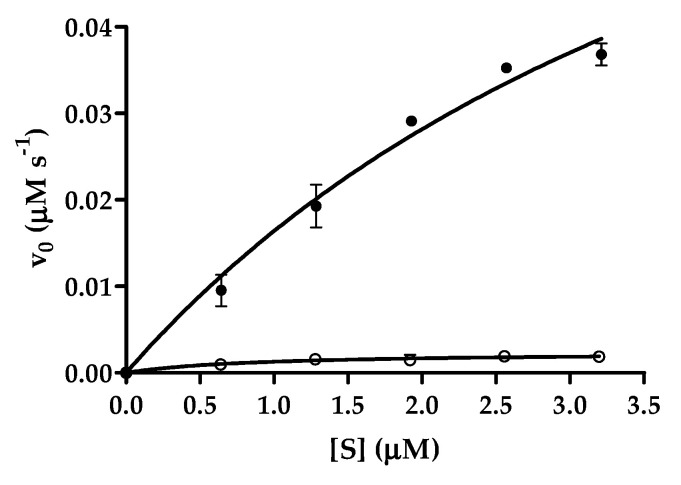
Kinetic analysis of SARS-CoV-2 3CLpro using recombinant fusion protein substrates. Data obtained for His_6_-MBP-TSAVLQ*SGFRKM-mEYFP and His_6_-MBP-REGTRVQ*SVEQIRE-mEYFP substrates are shown by full circles and squares, respectively. Averages of values obtained from two parallel experiments are plotted, error bars represent SD. Kinetic parameters are shown in Table 2.

After proving that the recombinant substrates are cleaved at the desired position (Figure 9), we performed cleavage reactions using the His_6_-MBP-mEYFP substrates to demonstrate that the fusion proteins are applicable for proteinase assays and to compare the cleavage efficiencies of SARS-CoV-2 and CTBP1 cleavage sites (Figure 10). 

Cleavage reaction revealed that His_6_-MBP-TSAVLQ*SGFRKM-mEYFP substrate containing a natural cleavage site of polyprotein is a better substrate of SARS-CoV-2 3CLpro as compared to the CTBP cleavage site-containing substrate (Figure 10) (Table 2). This is in agreement with the results of the gel-based assay which showed higher cleavage efficiency of His_6_-MBP-TSAVLQ*SGFRKM-mEYFP substrate as compared to His_6_-MBP-REGTRVQ*SVEQIRE-mEYFP (Figure 8) but is in contrast with the higher NetCorona score obtained for the latter cleavage site (Figure 3). Our results proved that the designed substrates can be used for proteolytic assays, and show that the substrate is processed at the incorporated CTBP1 only with low efficiency. 

The GTRVQ*SVEQI sequence motif is fully identical in the highly homologous CTBP1 and CTBP2 human proteins (Figure S2), therefore, CTBP2 is likely to be a target of SARS-CoV-2 3CL protease as well. In agreement with this, CTBP2 has been proved to be a proteolytic target of hCoV-NL63 3CLpro [21], and the highly similar cleavage site specificities imply that CTBP proteins may be potential targets of SARS-CoV and SARS-CoV-2 3CLpro enzymes, but their susceptibility for proteolytic cleavage needs to be investigated in the context of other cell types and/or species, as well.

## 3. Discussion

In this work, we aimed to test the application of such sequence-based algorithms for the prediction of SARS-CoV-2 3CLpro cleavage sites in different proteins which methods have already been applied in the case of SARS-CoV 3CLpro [26] or Zika and VEEV Group IV viral proteases [24,25].

Comparison of SARS-CoV and SARS-CoV-2 3CL proteases showed a high identity of protease sequences and substrate binding subsite compositions, which implied that the NetCorona v. 1.0 webserver—that has been developed for the prediction of SARS-CoV 3CLpro cleavage sites [26]—may be potentially applicable to identify potential host targets of SARS-CoV-2 3CLpro. In addition, identification of SSHHPS using BLAST analysis may be applied to identify putative target sequences of PLpro, however, prediction potential was not tested in this context. 

The most probable candidate host substrates were considered to contain SSHHPS and/or a potential cleavage site with a high NetCorona score, and of the candidate targets, we selected CTBP1 and PTK6 proteins and investigated their susceptibility for proteolysis in vitro. Additionally, IRAK1, which has already been predicted previously to contain a potential cleavage site of SARS-CoV 3CLpro [26], was also studied, and we proved that it is a substrate of SARS-CoV-2 3CLpro. Plasma protein PLMN—containing a predicted cleavage site—was not digested by SARS-CoV-2 3CLpro, possibly due to inaccessibility of the cleavage site in the structure.

A His_6_-MBP-TSAVLQ*SGFRKM-mEYFP recombinant substrate—containing a natural cleavage site of SARS-CoV-2 polyprotein—was designed and used as a positive control in cleavage reactions. This substrate system has already been applied previously to study proteases of HIV-1, tobacco etch virus [33,34,35], yeast Ty1 retrotransposon [36], and Venezuelan equine encephalitis virus (VEEV) [37], and the protease of human paternally expressed gene 10 (PEG10) protein [38]. The successful adaptation of this recombinant substrate system enables enzymatic characterization of SARS-CoV-2 3CLpro and screening of inhibitors using a microcentrifuge tube- [33,36] or a microtiter plate-based protease assay [34,37] in the future. Furthermore, a wide variety of sequences can be potentially inserted into recombinant substrates, making specificity studies and target site identifications possible. 

CTBP1 is a transcriptional co-repressor protein that is involved in the regulation of the expression of genes controlling development, oncogenesis, and apoptosis [39]. CTBP1 and -2 were found previously to influence viral replication, and enhanced replication of adenovirus E1A was observed upon CTBP knockdown [40]. PTK6 is also referred to as breast tumor kinase, and is an intracellular non-receptor tyrosine kinase, while PLMN is the zymogen form of plasmin being responsible for digestion of fibrin clot (fibrinolysis). Here we identified both proteins as candidate targets of SARS-CoV-2 3CLpro, but we did not observe their processing in spite of the presence of a putative cleavage site (predicted by the NetCorona webserver). 

The sets of experimentally determined cleavage sites—e.g., obtained from in vitro proteomic analysis [21]—are expected to aid the improvement of prediction algorithms’ reliability, while our results also represented some limitations of the applied in silico methods and highlighted the necessity of structural analysis and determination of cleavage site accessibilities, otherwise, candidate targets can be identified only with lower accuracy.

In summary, we have successfully adapted the SSHHPS analysis for the identification of potential coronavirus cleavage sites, and “repurposed” the NetCorona 1.0 webserver for the prediction of candidate human target proteins of SARS-CoV-2 3CLpro. We demonstrated that the NetCorona 1.0 webserver developed primarily for the 3CLpro of SARS-CoV is applicable efficiently for that of SARS-CoV-2, as well. The NetCorona webserver can be applied for the prediction of 3CLpro cleavage sites, while our results implied that SSHHPS analysis may be used to identify substrates of PLpro, as well, however, we have not tested PLpro in vitro. The prediction algorithms were tested only for human proteins, but they can be potentially adapted for the identification of host targets in other species as well. 

Our results highlighted a limitation of sequence-based cleavage site predictions and showed that the structural context of cleavage sites also need to be considered because the regions with the lower solvent-accessible surface may be less susceptible for proteolysis, even a high NetCorona score. We identified CTBP1 protein as a host substrate of SARS-CoV-2 3CLpro, and the existence of the predicted cleavage site was successfully proved experimentally both in the case of the recombinant CTBP1 and the His_6_-MBP-REGTRVQ*SVEQIRE-mEYFP substrate. Nonetheless, it is important to note that the CTBP cleavage site was processed with remarkably lower efficiency. Based on homology we assume that human CTBP2 is also a host substrate of the protease, but future studies need to reveal how processing of the CTBP proteins play role in the viral life-cycle. Identification of additional molecular targets of SARS-CoV and SARS-CoV-2 3CL proteases may help better understanding of viral replication, pathogenesis, and the coronavirus-induced phenotypes. 

## 4. Materials and Methods 

### 4.1. In Silico Analyses

#### 4.1.1. BLAST Analysis

Autoproteolytic cleavage site sequences of SARS-CoV-2 3CLpro were obtained from the literature [18]. BLAST analysis was performed to identify SSHHPS in human proteins, using the cleavage site sequences as input. Human-specific sequence search was run in BLASTP-as part of the BLAST+ 2.10.0—using the “blastp-short” option with PAM30 scoring matrix optimized for query sequences shorter than 30 residues [41,42]. The 10 residue-long query sequences (P5–P5’ residues) were aligned against the nr BLAST database (all non-redundant databases including GenBank translations, PDB, SwissProt, PIR, and PRF entries, excluding environmental samples from WGS projects) consisting of a total of 281,252,422 sequences. In order to include partially aligned hits of the catalytic residues, and those of similar physicochemical characteristics the following parameter values were set: window length, 15; cutoff value, 25,500; threshold score, 5.

#### 4.1.2. NetCorona Prediction

NetCorona v. 1.0 webserver (available at http://www.cbs.dtu.dk/services/NetCorona/) was applied to predict the presence of SARS-CoV-2 3CLpro cleavage sites [26], using sequences of full-length proteins as input. The default threshold of the NetCorona algorithm is 0.5. The higher prediction score indicates higher cleavage probability, the most probable cleavage sites were identified with > 0.8 prediction score.

#### 4.1.3. Structures

Coordinate files were downloaded from Protein Data Bank [43]. We used X-ray crystal structures of SARS-CoV-2 3CLpro (6LU7.pdb) [13], CTBP1 (4U6Q.pdb) [44], PLMN (1DDJ.pdb) [45], and ACHE proteins (1B41.pdb) [46], and a NMR solution structure of PTK6 (1RJA.pdb) [47]. The homology model of IRAK1 was downloaded from SWISS-MODEL Repository [48], the model has been prepared based on X-ray crystal structure of the protein (6BFN.pdb) [49].

The per-residue solvent accessible surface areas (SASA) were computed based on the coordinate files using Lee and Richard’s algorithm with default probe-radius (1.4 Å) at a resolution of 200 slices per atom by FreeSASA tool [50]. Structural figures were prepared by using the PyMol Molecular Graphics System (V. 1.3 Schrödinger, LLC). Sequence logos were prepared by the WebLogo 3 webserver [51].

### 4.2. In Vitro Analyses

#### 4.2.1. Materials

All materials were obtained from Sigma-Aldrich, otherwise, it is indicated. The purified recombinant human CTBP1 (ab93729), PTK6 (ab60888), and IRAK1 tagged with an N-terminal glutathione-S-transferase (ab268679) were ordered from Abcam, PLMN from Chromogenix, while BSA (A7030) from Sigma-Aldrich (St. louis, MO, USA). Lyophilized BSA and PLMN were dissolved in distilled water (1 mg/mL stock).

#### 4.2.2. Expression and Purification of SARS-CoV-2 3CLpro

A pET11a plasmid bearing the coding sequence of SARS-CoV-2 3CLpro (GenBank: MT291835.2) fused to an N-terminal hexahistidine tag (His_6_) was obtained using the gene synthesis service of GenScript. 

The pET11 bacterial expression plasmid coding for His_6_-SARS-CoV-2 3CLpro was transformed into BL21(DE3) *E. coli* cells. For protein expression, transformant cells were cultured at 37 °C in Luria-Bertani (LB) medium supplemented with ampicillin (100 µg/mL final concentration). Expression of the protease was induced by the addition of isopropyl β-D-1-thiogalactopyranoside (IPTG) in 1 mM final concentration, followed by shaking the suspensions at 37 °C for 3 h. After incubation, cells were collected by centrifugation at 4 °C for 20 min at 5000× *g* (Sorvall Lynx 4000, Thermo Fisher Scientific, Waltham, MA, USA). The pelleted cells were lysed in buffer A (20 mM Tris, 150 mM NaCl, 10 mM imidazole, pH 7.5) followed by sonication (Branson Sonifier 450) and centrifugation at 4 °C for 20 min at 10,000× *g*. His_6_-SARS-CoV-2 3CLpro was purified from the supernatant by a His-Trap Ni-chelate affinity chromatography column (GE Healthcare) using the Äkta Prime instrument (Amersham Pharmacia Biotech, Little Chalfont, UK). The buffer of the eluate was changed to buffer B (20 mM Tris, 1 mM DTT, pH 8.0) by ultrafiltration using Amicon Ultra centrifugal filters (10K, Merck Millipore, Burlington, MA, USA), followed by removal of His_6_-tag by digesting His_6_-SARS-CoV-2 3CLpro using Factor Xa (BCXA-1060, Haematologic Technologies, Essex Junction, VT, USA). Purification of the untagged protease was performed by ion-exchange chromatography using HiTrap Q FF column (GE Healthcare, Chicago, IL, USA). Finally, the fractions were dialyzed against buffer C (20 mM Tris, 150 mM NaCl, 1 mM EDTA, 1 mM DTT, pH 7.8), and stored at −20 °C. The purity of the fractions was determined by SDS-PAGE.

#### 4.2.3. Vector Construction for the Expression of a His_6_-MBP-mEYFP Substrates

The coding sequences of cleavage sites were cloned into a pDest-His_6_-MBP-mEYFP bacterial expression plasmid, based on the method described previously [33,34], the applied oligonucleotide primers and the cleavage site sequences are shown in Table 3. The success of cloning was confirmed by a DNA sequencing service (Eurofins Genomics Germany GmbH; Ebersberg, Germany), followed by a transformation of the verified pDest-His_6_-MBP-mEYFP expression constructs into BL21(DE3) *E. coli* cells.

#### 4.2.4. Expression and Purification of the His_6_-MBP-mEYFP Substrates

The His_6_-MBP-mEYFP protein substrates (Table 3) were expressed in BL21(DE3) *E. coli* cells based on the protocol described previously [33,34] with slight modifications. After expression at 37 °C, cells were collected by centrifugation, the pellet was suspended in lysis buffer (20 mM Tris HCl, 100 mM NaCl, 5 mM imidazole, pH 7.8), followed by sonication and centrifugation. The recombinant proteins were purified from the cleared cell lysates using Ni-NTA magnetic agarose beads (Cube Biotech, Germany) [33,34,35]. After purification, the elution buffer (100 mM EDTA, 0.05% Tween 20, pH 8.0) was exchanged for distilled water, and the total protein concentration was determined by measuring absorbance at 280 nm using NanoDrop 2000 (Thermo Fisher Scientific, Waltham, MA, USA). Sample purity was determined by SDS-PAGE, using 14% polyacrylamide gel. The purified fusion proteins were then used in cleavage reactions as substrates of SARS-CoV-2 3CLpro. 

#### 4.2.5. Cleavage Reactions by SARS-CoV-2 3CLpro

For cleavage reaction, the recombinant proteins were incubated with purified SARS-CoV-2 3CLpro in reaction buffer (20 mM Tris, 100 mM NaCl, pH 7.8) at 37 °C for at least 1 h. To analyze cleavage reactions by SDS-PAGE, the polyacrylamide gels were stained by Coomassie dye. In some cases, the denaturing SDS-PAGE was followed by in-gel renaturation of His_6_-MBP-mEYFP substrates by rinsing the gel in distilled water. The uncleaved substrate and cleavage products were visualized in the unstained gel based on their fluorescence under UV light using AlphaImager gel documentation system (ProteinSimple) [33,34,35], then the gel was stained by Coomassie dye as well. 

#### 4.2.6. Cleavage Site Identification by MALDI-TOF MS

For the identification of cleavage sites, the reaction mixtures were concentrated and desalted by using C4 ZipTip pipette tips (ZTC04S096, Sigma-Aldrich, St. Louis, MO, USA), based on the instructions of the manufacturer. 2,5-dihydroxybenzoic acid (DHB) (100 mg/mL) was applied as matrix dissolved in 50% aqueous acetonitrile with 0.1% TFA content. 0.5 µL matrix and 1 µL sample was deposited and mixed on the plate and was allowed to dry.

The mass spectrometric measurements were performed with a Bruker Autoflex Speed MALDI-TOF mass spectrometer. The linear mode was used for all samples, where the ion source voltage 1 and ion source voltage 2 were 19.5 kV, 18.3 kV, respectively. The applied laser was a solid phase laser (355 nm, ≥100 μJ/pulse) utilized at 200 Hz and 10,000 shots were summed. The results were evaluated by the flexAnalysis software (Bruker, Billerica, MA, USA).

#### 4.2.7. Proteinase Assay with His_6_-MBP-mEYFP Substrates

The magnetic bead-based assay was performed based on the method described previously [33,34,35,36] with slight modifications. Cleavage reactions were performed in reaction buffer (20 mM Tris, 100 mM NaCl, pH 7.8) by incubating samples at 37 °C for 10 min. For the measurements with His_6_-MBP-TSAVLQ*SGFRKM-mEYFP and His_6_-MBP-REGTRVQ*SVEQIRE-mEYFP substrates the enzyme was applied in 0.074 µM and 0.74 µM final concentration, respectively. Due to the lack of any selective tight-binding inhibitors, the determination of the active site concentration was not possible, and the activity of SARS-CoV-2 3CLpro was regarded as 100%. Fluorimetric measurements were performed using a Biotek Synergy H1 device at 510 nm excitation and 540 nm emission wavelengths. 

## Figures and Tables

**Table 1:** Prediction of cleavage sites of SARS-CoV-2 polyprotein by NetCorona 1.0 webserver. Sequence of SARS-CoV-2 polyprotein was used as input [31]. Values lower than the threshold (0.5) are not predicted as a potential cleavage site. Cleavage positions are shown by asterisks.

**SARS-CoV-2 Cleavage Site**	Protease	Sequence	NetCorona 1.0 Score
nsp1	PLpro	ELNGG*AYTRY	no score given
nsp2	PLpro	TLKGG*APTKV	no score given
nsp3	PLpro	ALKGG*KIVNN	no score given
nsp4	3CLpro	SAVLQ*SGFRK	0.891
nsp5	3CLpro	GVTFQ*SAVKR	0.458
nsp6	3CLpro	VATVQ*SKMSD	0.783
nsp7	3CLpro	RATLQ*AIASE	0.838
nsp8	3CLpro	AVKLQ*NNELS	0.860
nsp9	3CLpro	TVRLQ*AGNAT	0.904
nsp10	3CLpro	EPMLQ*SADAQ	0.865
nsp12	3CLpro	HTVLQ*AVGAC	0.905
nsp13	3CLpro	VATLQ*AENVT	0.680
nsp14	3CLpro	FTRLQ*SLENV	0.964
nsp15	3CLpro	YPKLQ*SSQAW	0.899

**Table 2:** Kinetic parameters determined for SARS-CoV-2 3CLpro using His_6_-MBP-TSAVLQ*SGFRKM-mEYFP and His_6_-MBP-REGTRVQ*SVEQIRE-mEYFP substrates. Data were calculated based on the results of two parallel experiments.

Cleavage Site	K_M_ (µM)	kcat (s^−1^)	kcat/K_M_ (µM^−1^s^−1^)
TSAVLQ*SGFRKM	5.086 ± 2.046	1.349 ± 0.370	0.2652 ± 0.1291
REGTRVQ*SVEQIRE	0.860 ± 0.303	0.0033 ± 0.0004	0.0038 ± 0.0014

**Table 3:** Oligonucleotide primers used for cloning. pDest-His_6_-MBP-mEYFP expression vectors were prepared by ligating the following complementary oligonucleotide primer pairs—coding for the cleavage site sequences-into the plasmid. CTBP1 (151–164) sequence is fully identical in CTBP1 and CTBP2 proteins. FW: forward; RV: reverse.

Cleavage Site	Sequence	Oligonucleotide Primer Sequence
SARS-CoV-2 nsp4	TSAVLQ* SGFRKM	FW: 5’-TAAAACCTCTGCGGTGCTGCAGTCTGGCTTTCGTAAAATGG-3’ RV: 5’-CTAGCCATTTTACGAAAGCCAGACTGCAGCACCGCAGAGGTTTTAAT-3’
CTBP1/2 (151–164)	REGTRV* SVEQIRE	FW: 5’-TAAACGTGAAGGCACCCGTGTGCAGTCTGTGGAACAGATCCGTGAAG-3’ RV: 5’-CTAGCTTCACGGATCTGTTCCACAGACTGCACACGGGTGCCTTCACGTTTAAT-3’

## Supplementary Materials

Supplementary materials can be found at https://www.mdpi.com/1422-0067/21/24/9523/s1.

## Abbreviations

3CLpro	3-chymotrypsin-like protease (main protease)
COVID-19	coronavirus disease-19
CTBP1	C-terminal-binding protein 1
CTBP2	C-terminal-binding protein 2
MBP	Maltose-binding protein
mEYFP	Monomeric enhanced yellow fluorescent protein
PLpro	Papain-like protease
PR	Protease
SARS-CoV-2	Severe acute respiratory syndrome coronavirus 2
SSHHPS	Short stretches of homologous host-pathogen protein sequences
