# Supplementary material for: Identification of Host Cellular Protein Substrates of SARS-COV-2 Main Protease
Source: Int J Mol Sci. 2020 Dec 15;21(24):9523. doi: 10.3390/ijms21249523 (PMC7765187; doi:10.3390/ijms21249523)
Supplement: Supplementary file 1 [file ijms-21-09523-s001.zip › Table_S2.docx]

**Table S2.** Prediction of SARS-CoV-2 3CLpro cleavage sites in plasma proteins. For prediction, NetCorona 1.0 web server was used, the putative cleavage site sequence and cleavage position is indicated for the predicted targets.

| **UniProt identifier** | **UniProt  entry name** | **Protein name** | **Predicted cleavage** | **Prediction score** | **Cleavage position** | **Cleavage site sequence** |
| --- | --- | --- | --- | --- | --- | --- |
| P02768 | ALBU_HUMAN | Human serum albumin | no | - | - | - |
| P02769 | ALBU_BOVIN | Bovine serum albumin | no | - | - | - |
| P02671 | FIBA_HUMAN | Human fibrinogen alpha chain | no | - | - | - |
| P02675 | FIBB_HUMAN | Human fibrinogen beta chain | no | - | - | - |
| P02679 | FIBG_HUMAN | Human fibrinogen gamma chain | no | - | - | - |
| P00747 | PLMN_HUMAN | Human plasminogen | yes | 0.649 | 740 | NGRVQ*STELC |
| P00749 | UROK_HUMAN | Human urokinase-type plasminogen activator | no | - | - | - |
| P00750 | TPA_HUMAN | Human tissue-type plasminogen activator | no | - | - | - |
| P05120 | PAI2_HUMAN | Human plasminogen activator inhibitor 2 | yes | 0.589 | 96 | DAILQ*AQAAD |
| P05121 | PAI1_HUMAN | Human plasminogen activator inhibitor 1 | no | - | - | - |
