# Supplementary material for: Identification of Host Cellular Protein Substrates of SARS-COV-2 Main Protease
Source: Int J Mol Sci. 2020 Dec 15;21(24):9523. doi: 10.3390/ijms21249523 (PMC7765187; doi:10.3390/ijms21249523)
Supplement: Supplementary file 1 [file ijms-21-09523-s001.zip › Figure_S2.docx]

**Figure S2.** Sequence alignment of human CTBP1 and CTBP2 proteins. Sequence identifiers are shown for both proteins. Cleavage site predicted by NetCorona web server is shown by green background (P5-P5’ residues).

Q13363|CTBP1_HUMAN ------MGSSHLLNKGLPLGVRPPIMNGPLHPRPLVALLDGRDCTVEMPILKDVATVAFC 54

P56545|CTBP2_HUMAN MALVDKHKVKRQRLDRICEGIRPQIMNGPLHPRPLVALLDGRDCTVEMPILKDLATVAFC 60

.: . : *:** *****************************:******

Q13363|CTBP1_HUMAN DAQSTQEIHEKVLNEAVGALMYHTITLTREDLEKFKALRIIVRIGSGFDNIDIKSAGDLG 114

P56545|CTBP2_HUMAN DAQSTQEIHEKVLNEAVGAMMYHTITLTREDLEKFKALRVIVRIGSGYDNVDIKAAGELG 120

*******************:*******************:*******:**:***:**:**

Q13363|CTBP1_HUMAN IAVCNVPAASVEETADSTLCHILNLYRRATWLHQALREGTRVQSVEQIREVASGAARIRG 174

P56545|CTBP2_HUMAN IAVCNIPSAAVEETADSTICHILNLYRRNTWLYQALREGTRVQSVEQIREVASGAARIRG 180

*****:*:*:********:********* ***:***************************

Q13363|CTBP1_HUMAN ETLGIIGLGRVGQAVALRAKAFGFNVLFYDPYLSDGVERALGLQRVSTLQDLLFHSDCVT 234

P56545|CTBP2_HUMAN ETLGLIGFGRTGQAVAVRAKAFGFSVIFYDPYLQDGIERSLGVQRVYTLQDLLYQSDCVS 240

****:**:**.*****:*******.*:******.**:**:**:*** ******::****:

Q13363|CTBP1_HUMAN LHCGLNEHNHHLINDFTVKQMRQGAFLVNTARGGLVDEKALAQALKEGRIRGAALDVHES 294

P56545|CTBP2_HUMAN LHCNLNEHNHHLINDFTIKQMRQGAFLVNAARGGLVDEKALAQALKEGRIRGAALDVHES 300

***.*************:***********:******************************

Q13363|CTBP1_HUMAN EPFSFSQGPLKDAPNLICTPHAAWYSEQASIEMREEAAREIRRAITGRIPDSLKNCVNKD 354

P56545|CTBP2_HUMAN EPFSFAQGPLKDAPNLICTPHTAWYSEQASLEMREAAATEIRRAITGRIPESLRNCVNKE 360

*****:***************:********:**** ** ***********:**:*****:

Q13363|CTBP1_HUMAN HLTAATHWASMDPAVVHPELNGAAYRYPPGVVGVAPTGIPAAVEGIVPSAMSLSHGLPPV 414

P56545|CTBP2_HUMAN FFVTSAPWSVIDQQAIHPELNGATYRYPPGIVGVAPGGLPAAMEGIIPGGIPVTHNLPTV 420

.:.::: *: :* .:*******:******:***** *:***:***:*..: ::*.** *

Q13363|CTBP1_HUMAN AHPPHAPSPGQTVKPEADRDHASDQL 440

P56545|CTBP2_HUMAN AHPSQAPSPNQPTKHGDNREHPNEQ- 445

*** :****.* .* :*:* .:*
