# Supplementary material for: Identification of Host Cellular Protein Substrates of SARS-COV-2 Main Protease
Source: Int J Mol Sci. 2020 Dec 15;21(24):9523. doi: 10.3390/ijms21249523 (PMC7765187; doi:10.3390/ijms21249523)

**Figure S1.** Prediction of SARS-CoV-2 3CLpro cleavage sites in CTBP1. Sequence of human CTBP1 protein was used as input (Q13363; CTBP1_HUMAN) in NetCorona web server, predicted 3CLpro cleavage site is highlighted by red. A potential target sequence of PLpro identified by SSHHPS analysis is also shown by blue background.


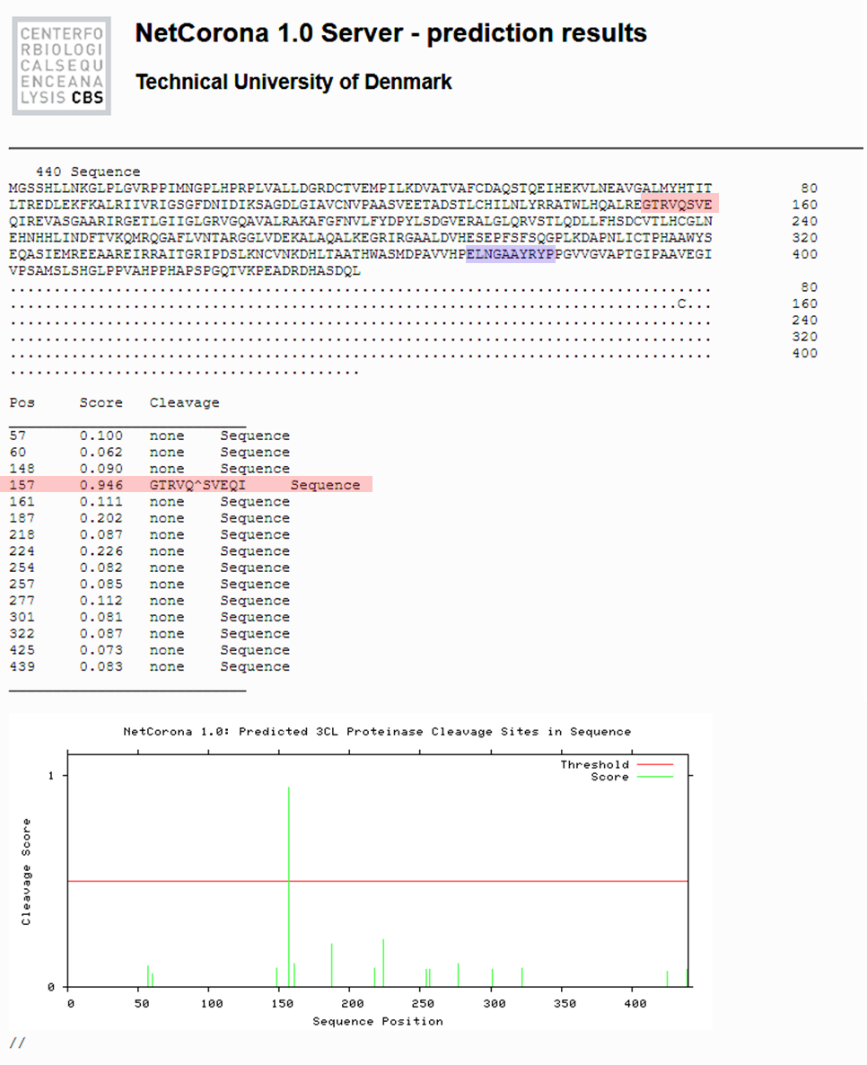

Supplement: Supplementary file 1 [file ijms-21-09523-s001.zip › Figure_S1.docx]
